# Behçet’s disease: new insight into the relationship between procoagulant state, endothelial activation/damage and disease activity

**DOI:** 10.1186/1750-1172-8-81

**Published:** 2013-05-27

**Authors:** Ihosvany Fernández-Bello, Francisco J López-Longo, Elena G Arias-Salgado, Víctor Jiménez-Yuste, Nora V Butta

**Affiliations:** 1 Hematology Unit, Hospital Universitario La Paz-IdiPAZ, Madrid, Spain; 2 Rheumatology Unit, Hospital Universitario Gregorio Marañón, Madrid, Spain; 3 Universidad Autónoma de Madrid, Madrid, Spain

**Keywords:** Behçet, E-selectin, PAI-1, Fibrinogen, Thromboelastometry, Thrombin generation, Prothrombotic state

## Abstract

**Background:**

Behçet disease (BD) is associated with a prothrombotic state of unknown origin that may lead to life-threatening events. Calibrated Automated Thrombogram (CAT) and Rotational Thromboelastometry (ROTEM) are two global haemostasis assays that may reveal new insights into the physiopathological mechanisms of the disease and its procoagulant condition.

**Methods:**

23 BD patients who had no signs or symptoms of current thrombosis and 33 age- and sex-matched controls were included in the study. We performed ROTEM and CAT tests and assessed erythrocyte count, platelet count, platelet contribution to clot formation and plasma levels of tissue-type plasminogen activator, plasminogen activator inhibitor type 1 (PAI-1), fibrinogen, C-reactive protein (CRP), thrombin-antithrombin III complex (TAT), D-dimer and E-selectin (ES).

**Results:**

Both ROTEM and CAT tests showed a hypercoagulable state in the BD patients. Plasma levels of PAI-1, fibrinogen, TAT, CRP and ES were significantly increased in this group compared to controls. The disease activity (DA) was significantly correlated with levels of ES and the maximum clot firmness, and this last one, in turn, correlated with rising levels of ES, PAI-1, CRP and fibrinogen. CAT parameters did not correlate with DA or ES.

**Conclusions:**

Both ROTEM and CAT tests reveal that patients with BD have a procoagulant state even in the absence of thrombosis. ROTEM test indicates that increased levels of fibrinogen and PAI-1 may be involved in the prothrombotic state of this pathology, while platelets do not significantly contribute. Moreover, CAT assay demonstrate that plasma from BD patients is able to generate more thrombin than controls in response to the same stimulus and that this effect is independent of the DA and the endothelial impairment suggesting the involvement of another factor in the hypercoagulable state observed in BD patients. This study also shows that endothelium activation/damage may be a contributing factor in both the procoagulant and clinical conditions of BD, as shown by the direct correlation between ES levels, ROTEM parameters and DA.

## Introduction

Behçet’s disease (BD), also known as Adamantiades–Behçet's disease, is a rare multisystem, inflammatory disease of unknown aetiology [1] and is associated with thrombogenicity [2]. Gender distribution and clinical expression of BD varies with ethnic origin and seems to depend on the geographic area of the BD patients’ residence [3].

The diagnosis of BD is primarily based on clinical manifestations. New international criteria [3] have recently been published as diagnostic criteria for BD and include vascular manifestations, which is of great importance due to the frequency of serious vascular and large vessel involvement as has been recently reported [4]. However, patients who were diagnosed according to the International Study Group Criteria (1990) [5] also meet the new International Criteria (2013).

The pathogenesis of the prothrombotic state in BD is not known, although associated vascular damage/activation seems to be an important key factor [6,7]. Increased levels of several procoagulant markers [6,8], defective fibrinolysis [8-11], and altered platelet function [12,13] are some of the numerous findings that have been obtained to date; however, these variables have been investigated individually using various methods and, in some cases, have led to conflicting results.

In recent years, there has been growing interest in the use of global coagulation tests in the evaluation of bleeding diathesis and the hypercoagulable state. Rotational thromboelastometry (ROTEM) is a viscoelastometric clotting test that measures the kinetics of clot formation and fibrinolysis, providing global information on the cellular and soluble procoagulant/anticoagulant protein interactions. Calibrated automated thrombogram (CAT) is a thrombin generation test developed by Hemker *et al.*[14] that quantifies thrombin generation beyond the end points of traditional coagulation tests. Both techniques have been applied successfully to the study of the procoagulant state associated with various conditions, including inflammatory states [15-17].

Considering that haemostasis emerges from the interplay between different cells, coagulation factors and inhibitors, these global tests may reveal a better understanding of the hypercoagulable condition in BD than has been obtained so far through subrogated and isolated procoagulant markers. Therefore, we investigated the potential utility of ROTEM and CAT in the characterisation of the procoagulant state in BD and assessed whether these tests offer a new insight into the physiopathological mechanisms of the disease and its procoagulant profile.

## Methods

### Study design and subjects

This was a case–control study. Patients diagnosed with BD, according to the criteria of the *International Study Group for Behçet’s Disease*[5], who were over 18 years of age and who attended the Rheumatology Unit of the Gregorio Marañón University Hospital were invited to participate in the study. The activity index was determined as described by the Bhakta *et al.* guidelines [18]. Healthy blood donors from the blood donation centre of the La Paz University Hospital were included as controls. The hospital ethic committee approved the experimental protocol, and subjects were included after signing the informed consent. Exclusion criteria were smoking, the use of oral contraceptives, anticoagulant or active antiplatelet drugs, uncontrolled hypertension, diabetes, hyperlipidaemia, peripheral or coronary artery disease, the presence of abnormal hepatic or renal function, antiphospholipid antibodies (anti-cardiolipin, anti-beta2-glycoprotein I and lupus anticoagulant) and lupus.

### Collection and handling of samples

To avoid the influence of circadian changes in the study variables, the collection time was the same for all subjects (between 9 A.M. and 10 A.M.). Peripheral blood was collected in ethylenediaminetetraacetic acid tubes (Becton, Dickinson and Company, Madrid, Spain) for blood cell count and in sodium citrate (1.3%) tubes (Becton, Dickinson and Company, Madrid, Spain) for the remaining tests. Citrated whole blood was centrifuged at 2500 g for 20 min at 23°C to obtain platelet poor plasma (PPP). PPP aliquots were stored immediately at −70°C until analysis. All samples were analysed or stored properly within two hours of sampling.

### Calibrated Automated Thrombogram (CAT)

Thrombin generation was measured in PPP by CAT as described previously [14]. All measurements were performed after 10 minutes of preheating at 37°C. Coagulation was triggered by proper recalcification and the addition (final concentrations) of 1 pM of recombinant human tissue factor and 4 μM of phospholipid mixture (PPP-Reagent LOW, Thrombinoscope BV, Maastricht, The Netherlands). Lag time (LT, time required for the formation of 10 nM thrombin), time-to-peak (TTP, time required to reach the maximum thrombin concentration), peak height (PH, maximum thrombin concentration achieved), and endogenous thrombin potential (ETP, area under the thrombin concentration vs. time curve) were calculated with the Thrombinoscope software package (Thrombinoscope BV, Maastricht, The Netherlands). The velocity index (VI), a parameter related to the speed with which thrombin is produced, was calculated from the experimental data as follows:

$$\mathrm{VI}=\frac{\mathrm{PH}}{\left(\mathrm{TTP}-\mathrm{LT}\right)}$$

### Rotational Thromboelastometry (ROTEM)

ROTEM was performed on whole blood that was allowed to rest at room temperature for 30 min before testing. A partial thromboplastin phospholipid and ellagic acid-activated intrinsic pathway (INTEM test) was performed to assess the kinetics of clot formation. We recorded the clot formation time (INTEM-CFT, time to 20-mm amplitude [in seconds], which reflects the speed of the clotting process), alpha angle (INTEM-α, tangent to the curve at 2-mm amplitude [in degrees], which reflects the rate of fibrin polymerisation), and maximum clot firmness (INTEM-MCF, maximum clot firmness [in mm], which reflects the maximum tensile strength of the thrombus). To assess the contribution of platelets to the clot kinetics, a platelet-inhibited FIBTEM test was performed and compared with the INTEM test for MCF using the following formula:

$$\begin{array}{l} \mathrm{Platelet}\;\mathrm{contribution}\left(\mathrm{MCF}\right)\\ \;=\frac{\left[\mathrm{INTEM}\left(\mathrm{MCF}\right)-\mathrm{FIBTEM}\left(\mathrm{MCF}\right)\right]}{\mathrm{INTEM}\left(\mathrm{MCF}\right)}\times 100 \end{array}$$

### Cell count, biochemistry and study of fibrinolysis

The blood cell count was performed with a Coulter AcT Diff cell counter (Beckman Coulter, Madrid, Spain). Plasma levels of D-dimer (DD) and fibrinogen were determined using a BCS^©^ XP system (Siemens Healthcare Diagnostics Products GmbH, Marburg, Germany) and C-reactive protein (CRP) was measured by nephelometric method (Behring Nephelometer Analyzer, Germany). Thrombin-antithrombin III complex (TAT) (AssayMax Human Thrombin-antithrombin Complexes ELISA Kit, ASSAYPRO, St. Charles, MO, USA) and E-selectin (ES) (Human sE-Selectin/CD26E Immunoassay Quantikine^©^ ELISA kit, R&D Systems Europe Ltd., Abingdon, UK) were measured in PPP, following the manufacturer’s instructions. The fibrinolytic profile was evaluated by assessing plasma antigenic levels of tissue-type plasminogen activator (t-PA) (TriniLIZE tPA antigen ELISA kit) and plasminogen activator inhibitor type 1 (PAI-1) (TriniLIZE PAI-1 antigen ELISA kit); all kits were acquired from Trinity Biotech, Bray, Co Wicklow, Ireland.

### Statistical analysis

The results are expressed as the mean ± SD, the median and range (25^th^-75^th^ percentile) or as the absolute value (percentage of the total). We performed an unpaired Student's *t*-test and the Mann–Whitney *U* test as needed to compare variables between the groups. The associations between the variables were calculated using Pearson’s or Spearman's correlation test, depending on the data distribution. Normality was tested by a Shapiro-Wilk test. Statistical analyses were performed using SPSS software version 17.0 for Windows (SPSS, Chicago, IL, USA). Values of P ≤ 0.05 were considered statistically significant.

## Results

Of the 33 unrelated BD patients interviewed, 23 were included and compared with 33 age and gender matched healthy subjects. Ten patients were excluded because they did not fulfil inclusion criteria. None of the interviewed patients had signs or symptoms of current thrombosis. The clinical and treatment characteristics of the patients are summarised in Table 1.

**Table 1:** Clinical features and treatment of BD patients at the time of the study and cumulative

**Characteristic**	**At time of the study**	**Cumulative**
Clinical features: N = 23		
Disease duration in years (mean ± SD)	15 ± 8	-
Deep vein thrombosis (%)	0	4
Disease activity, median (p25-p75)	7 (2–18)	-
Patients on active phase of disease (%)	78	-
Genital ulcer (%)	13	97
Oral ulcer (%)	52	100
Skin lesion (%)	43	90
Vascular involvement (%)	0	21
Articular involvement (%)	43	67
Gastrointestinal involvement (%)	30	32
Ocular involvement (%)	22	64
Neurological involvement (%)	43	72
Treatments:		
Prednisone (< 10 mg) (%)	9	83
Prednisone (> 10 mg) (%)	18	87
Cyclosporine (%)	4	18
Azathioprine (%)	9	32
Methotrexate (%)	4	7
Infliximab (%)	4	3
Rituximab (%)	4	7
Colchicine (%)	17	86

### Cell count, biochemistry and study of fibrinolysis

We found significantly increased levels of fibrinogen, CRP, PAI-1 antigen, TAT and ES in the BD patients (Table 2). There were no significant differences in the other variables between the groups (Table 2).

**Table 2:** Demographic features, ROTEM and CAT results of both groups

**Characteristics**		**BD patients (N = 23)**	**Controls (N = 33)**	**p-value**
Age at inclusion (year)		49 ± 15	43 ± 10	0.082
Female gender (%)		78	63	0.241
	INTEM-α (degree)	77 ± 3	74 ± 2	<0.010
ROTEM	INTEM-CFT (sec)	66 ± 15	80 ± 14	<0.010
	INTEM-MCF (mm)	61 ± 4	57 ± 4	<0.010
	Platelet contribution-MCF (%)	78 (69–79)	79 (77–81)	0.090
	Lag-time (min)	6.7 ± 1.8	5.6 ± 1.1	0.025
CAT	Time-to-peak (min)	9.6 ± 1.9	9.0 ± 1.4	0.240
	Peak height (nM)	292 ± 65	213 ± 67	<0.010
	ETP (nM × min)	1543 ± 331	1286 ± 292	0.010
	Velocity index (nM/min)	102 (83–127)	55 (42–90)	<0.010
	Platelet count (×10^3^/μl)	217 (189–287)	227 (199–247)	0.785
	Erythrocyte count (×10^6^/μl)	4.2 ± 0.4	4.3 ± 0.4	0.244
	Fibrinogen (mg/l)	321 ± 53	280 ± 42	<0.010
	TAT (ng/l)	19 (17–21)	15 (12–17)	<0.010
Other parameters	D-dimer (μg/l)	293 ± 65	270 ± 86	0.480
	tPA (antigen) (ng/ml)	17.2 ± 7.2	12.4 ± 5.2	0.120
	PAI-1 (antigen) (ng/ml)	19.9 ± 8.6	12.6 ± 6.0	0.032
	E-selectin (μg/l)	24.2 ± 9.3	16.8 ± 8.4	0.040
	C-reactive protein (mg/dl)	0.2 (0.1-0.5)	0.1 (0.1-0.4)	0.046

Results are expressed as the mean ± SD, the median and range (25^th^-75^th^ percentile) or as absolute value (%).

### Rotational Thromboelastometry (ROTEM)

The coagulation profiles assessed by the ROTEM test showed enhanced coagulation in patients with BD. The clot formation speed (evaluated by INTEM-α and INTEM-CFT) and the INTEM-MCF were significantly higher in this group (Table 2). INTEM-MCF showed correlations with fibrinogen levels in both patients and controls (patients: r = 0.620, p = 0.005; controls: r = 0.638, p = 0.002) and with CRP in the BD group (r = 0.384, p = 0.02). INTEM-MCF, INTEM-α and INTEM-CFT were affected by the levels of PAI-1 (INTEM-MCF: r = 0.648, p = 0.012; INTEM-α: r = 0.650, p = 0.012 and INTEM-CFT: r = −0.690, p = 0.006) in the BD patients. In controls, INTEM-CFT correlated with the levels of PAI-1 (r = 0.645, p = 0.044). The calculated percentage of platelet contribution to INTEM-MCF was similar in both groups, suggesting a poor contribution of platelets to the procoagulant state observed by thromboelastometry in the BD patients (Table 2).

### Calibrated Automated Thrombogram (CAT)

LT, PH, ETP and VI were significantly increased in patients with BD (Table 2). In this group, the levels of fibrinogen correlated with LT (r = 0.622, p = 0.004), TTP (r = 0.548, p = 0.015) and ETP (r = 0.452, p = 0.05). We found a moderate negative correlation between LT and INTEM-CFT (r = −0.568, p = 0.007) and a moderate positive correlation between LT and INTEM-α (r = 0.536, p = 0.012). This provided compelling evidence that the increased thrombus formation in BD patients was associated with a delay in the start of thrombin generation in the CAT test. However, we observed a positive dependence between PH and the rate of fibrin polymerisation (INTEM-α: r = 0.432, p = 0.05), which suggests that the increase in the clot formation speed might be related with the higher thrombin generation capacity in this group.

### Relationship between DA, coagulation status and endothelial cell injury/activation

**Figure 1:**
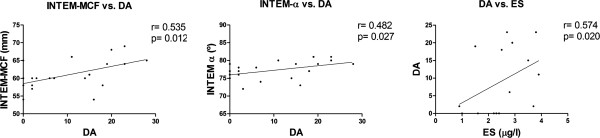
Correlation between disease activity (DA) and INTEM-MCF, INTEM-α and soluble E-selectin (ES).

**Figure 2:**
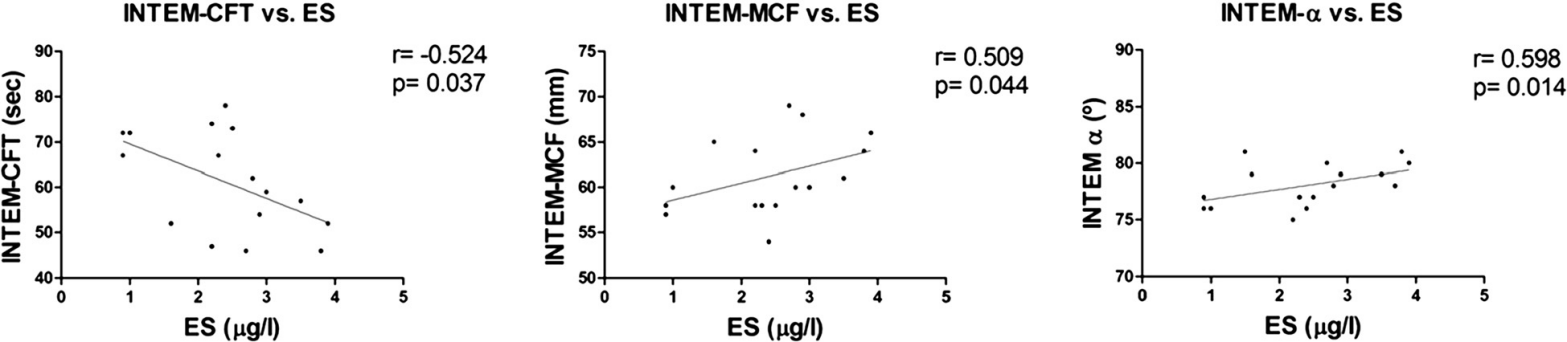
Correlation between soluble E-selectin (ES) and INTEM-CFT, INTEM-MCF and INTEM-α.

We observed correlations between DA and INTEM-MCF, INTEM-α and ES levels (Figure 1), indicating that increased DA was associated with a higher thrombus formation capacity and more severe vascular injury/activation. In turn, the ES levels correlated with INTEM-CFT, INTEM-MCF and INTEM-α (Figure 2), suggesting a possible relationship between endothelial cell injury/activation and the hypercoagulable state observed by the ROTEM test in the BD patients. Except for the fibrinogen and INTEM-MCF, there were no correlations between ROTEM parameters and the other variables in the control group.

We also observed a significant correlation between antigenic levels of PAI-1 and DA (r = 0.739, p = 0.009) and ES levels (r = 0.640, p = 0.034) in patients with active disease (DA > 0), which implicates PAI-1 as a potential link between vasculitis and/or the inflammatory state and enhanced clot formation in BD patients. The CAT parameters failed to correlate with ES levels and DA in the BD patients.

## Discussion

As far as we know, this is the first report on the use of ROTEM and CAT in the study of the procoagulant state in BD. The present study has shown that thrombin generation and blood clotting capacity were increased in BD patients, even in the absence of thrombosis. Additionally, we found significant correlations between the ROTEM parameters, plasma ES levels and DA, which suggests links between the procoagulant state, endothelial inflammation and severity of symptoms of the disease.

We found increased levels of fibrinogen in our group of patients with BD. This result is consistent with those observed in previous studies [19-21] and is not surprising considering that 78% of our patients were in an active state and that fibrinogen is an acute phase reactant [22]. The procoagulant effect of fibrinogen on ROTEM parameters has been reported previously [23], and our results are in agreement with this effect. We observed that fibrinogen levels were significantly correlated with INTEM-MCF in both patients and controls, which suggests that increased levels of fibrinogen in the BD group might be involved in the group’s hypercoagulable profile, as determined by the ROTEM test. Surprisingly, although INTEM-MCF correlated significantly with the fibrinogen levels and DA, we did not observe any dependence between the fibrinogen levels and DA. This issue, which was also observed by Hampton *et al.*[19], is intriguing but may be the result of the high variability of the fibrinogen synthesis route due to alternative mRNA processing, post-translational modifications and proteolytic degradation, which may lead to different levels of fibrinogen in response to similar inflammatory states [24]. Fibrinogen may also affect thrombin generation. Dielis *et al.* have shown that fibrinogen may produce not only an anticoagulant effect by increasing LT but also a procoagulant effect by heightening ETP and PH values in normal populations [25]. This dual anticoagulant/procoagulant effect can be explained by the spatial distribution of the thrombin’s binding sites and the kinetics of interaction with its multiple substrates. Increased fibrinogen levels may prolong LT because of this molecule’s ability to bind to thrombin through exosite II, which is needed for the thrombin-mediated FVIII activation. This fibrinogen binding leads to an anticoagulant effect in the initiation phase at low tissue factor concentrations [26,27]. This evidence has also been supported by Hemker *et al.* who compared thrombin generation in full and defibrinated plasma [28]. It is interesting to mention that a prolonged LT despite an increased thrombotic risk is observed in patients with antiphospholipid syndrome [29] which suggests that an extended LT does not rule out the presence of a prothrombotic state. Fibrinogen may also increase thrombin generation due to fibrin’s ability to protect thrombin from inhibition by antithrombin III [28,30]. Our results agree with these hypotheses, given that we found a significant positive correlation between fibrinogen levels and ETP in patients with BD. In summary, although fibrinogen could induce an anticoagulant effect by increasing the LT for generating thrombin, the amount of thrombin generated (ETP) and the strength of the clot (INTEM-MCF) were increased by higher levels of fibrinogen in the BD group. This condition could be responsible, at least in part, for the procoagulant pattern observed in the BD patients by the two global tests.

Previous studies have reported a correlation between platelet count (PTS) and clot formation speed and strength [31]. However, we did not find any differences in PTS between the patients and the controls. Although higher platelet activation and response to stimulus have been reported in BD patients [13,32], our research group recently published a study of platelet function in the same group of BD patients included in the present study and found no differences in platelet activation markers between the controls and the BD patients, either at baseline conditions or after stimulation with agonists (TRAP and ADP) [33]. After considering this finding and the lack of differences in PTS and platelet contribution to the ROTEM trace between the groups, we conclude that platelets are not the cause of the deviation in the ROTEM results.

A study by Spiezia *et al*. (2008) suggests that erythrocyte count (ERY) may decrease clot firmness [34]. In the present study, we did not observe any significant differences in the ERY between the patients and controls, and therefore the contribution of ERY to the differences observed in the ROTEM trace between the two groups seems negligible.

It has been reported that abnormal fibrinolysis may contribute to thrombosis, atherosclerosis and vascular stenosis [35]. Fibrinolysis has been previously studied in BD with conflicting results, which was probably due to the high variability of patients included in the studies and the different assays used in each case [8-10,19,36-38]. In the present study, BD patients showed tPA antigen levels similar to controls but had significantly increased PAI-1 antigen plasma levels, suggesting a possible hypofibrinolitic profile in this group. Systemic inflammation as presented in BD could increase PAI-1 levels [39,40]. Moreover, it has been reported that platelet stimulation by thrombin induces platelet synthesis and release of active PAI-1 [41,42] and, in fact, increased platelet activation has been highly correlated to plasma PAI-1 activity in acute stroke patients [43]. Thrombin may also stimulate platelet synthesis and secretion of TGF beta [44-46]. TGF beta, in turn, may increase the synthesis of PAI-1 in endothelial cells [44-46]. These mechanisms may explain, at least in part, the increased plasma levels of PAI-1 in BD patients because they show systemic activation of coagulation and increased thrombin production in response to stimulus. Increased levels of PAI-1 can increase the clot formation speed and clot stability [47,48] due to the rapid and irreversible blockage of the protease activity of tPA, the main plasminogen activator [49]. Our results agree with this observation given that we found a significant correlation between antigenic levels of PAI-1 and INTEM-CFT, INTEM-α and INTEM-MCF, which points to PAI-1 as a key factor in the procoagulant state observed in BD patients by this test. In spite of the fact that an association between levels of PAI-1 and thrombosis in BD has not been reported, relief from vascular events and oral ulcers after treatment with profibrinolytic agents has been observed in these patients [50,51]. Moreover, we and other groups have observed a positive correlation between PAI-1 levels and DA, suggesting a probable association between the impaired fibrinolysis in BD and the severity of the disease symptoms [19,52]. Whether this finding reflects a causal relation between BD symptoms and defective fibrinolysis is an issue that needs to be evaluated in further studies with larger numbers of patients.

The procoagulant state observed by the CAT and ROTEM tests in the BD patients was supported by the increase in plasma TAT, a marker of intravascular thrombin formation. However, the TAT level did not correlate to the ROTEM and CAT parameters. A lack of correlation between TAT levels and CAT and ROTEM values has been previously reported [53-55] which suggests that the TAT level might indicate that activation of coagulation had occurred but does not necessarily reflect the patient’s procoagulant potential at the time of the sampling.

In contrast to previous reports that indicated high DD levels in BD patients with active disease and deep vein thrombosis [11], we did not find any differences in DD between the BD patients and controls. This controversial result may be due to the absence of signs, symptoms or recent history of thrombosis in our patients.

Endothelial damage has been described as a potential key factor involved in the prothrombotic state of BD [7,8], and ES, a marker of endothelial damage/activation, has been found to be increased in the active state of the disease [56]. Our results were in correspondence with this data as we found higher levels of ES in the BD patients compared with controls that correlate with DA (Figure 1). When analyzing the correlation between ES and ROTEM and CAT parameters, we found a significant correlation between ES levels and the ROTEM parameters but not between ES levels and the CAT parameters. We also failed to obtain any correlation between CAT parameters and DA that by the contrary showed correlation with the procoagulant profile observed by the ROTEM test. One hypothesis to explain this effect may be based on the fact that the CAT test is only able to depict the thrombin generation capacity of the plasma, whereas the ROTEM test describes thrombin generation, clot formation and fibrinolysis. As shown above, these processes may be altered in this disease, and therefore the ROTEM test may be a more appropriate test for describing the associated endothelial and inflammatory pathological condition of the disease.

Our results support the existence of an increased procoagulant state in BD patients, and they raise the question about the usefulness of anticoagulant treatments in these patients. This is a matter of controversy as there is no consensus among rheumatologists from various countries [57] because lifelong anticoagulation treatment might increase the risk of fatal bleeding [58]. Moreover, given that blood vessel wall inflammation is a possible cause of thrombosis in BD patients, the use of immunosuppressive therapy might be a more rational choice [59,60].

## Conclusions

The ROTEM test is a useful tool for studying of hypercoagulable state in BD. Additionally, CAT experiments reveal that plasma from BD patients is able to produce faster and higher thrombin generation. Our data also indicated that endothelial activation/damage is involved in both the clinical manifestation and procoagulant state of this pathology. Moreover, the heightening of fibrinogen and PAI-1 may be important components in the procoagulant condition of the disease, whereas the effect of platelets seemed to be almost negligible. Further studies are warranted to evaluate the relationship between PAI-1 levels and the symptoms of the disease and to determine whether the ROTEM test and ES levels are useful tools/markers for monitoring therapeutic response and disease progression in BD patients.

## Abbreviations

BD: Behçet disease; CAT: Calibrated automated thrombogram; ROTEM: Rotational thromboelastometry; PAI-1: Plasminogen activator inhibitor type 1; tPA: tissue-type plasminogen activator; TAT: Thrombin-antithrombin III complex; DD: D-dimer; ES: E-selectin; DA: Disease activity; PPP: Platelet poor plasma; LT: Lag time; TTP: Time-to-peak; PH: Peak height; ETP: Endogenous thrombin potential; VI: Velocity index; CFT: Clot formation time; a: Alpha angle; MCF: Maximum clot firmness; PTS: Platelet count; ERY: Erythrocyte count.

## Competing interests

The authors declare that they have no competing interests.

## Authors’ contributions

NVB was the principal investigator, coordinated the group, obtained the funding for the study and participated in study design, statistical analysis and writing of paper. IFB carried out the CAT and ROTEM experiments and participate in study design, statistical analysis and writing of paper. FJLL recruited the patients, participated in the design of the study, the analysis of the data and helped to draft the manuscript. EGAS: carried out the immunoassays. VJY: participated in the analysis of the data. All authors read and approved the final manuscript.
